# High-throughput sequencing for community analysis: the promise of DNA barcoding to uncover diversity, relatedness, abundances and interactions in spider communities

**DOI:** 10.1007/s00427-020-00652-x

**Published:** 2020-02-10

**Authors:** Susan R. Kennedy, Stefan Prost, Isaac Overcast, Andrew J. Rominger, Rosemary G. Gillespie, Henrik Krehenwinkel

**Affiliations:** 1 Biodiversity and Biocomplexity Unit, Okinawa Institute of Science and Technology, Onna, Okinawa, Japan; 2 LOEWE-Centre for Translational Biodiversity Genomics, Senckenberg Museum, Frankfurt, Germany; 3 National Zoological Garden, South African National Biodiversity Institute, Pretoria, South Africa; 4 Graduate Center of the City University New York, New York, NY USA; 5 Ecole Normale Supérieure, Paris, France; 6 Santa Fe Institute, Santa Fe, NM USA; 7 Environmental Sciences Policy and Management, University of California Berkeley, Berkeley, CA USA; 8 Department of Biogeography, Trier University, Trier, Germany

**Keywords:** Metabarcoding, Portable sequencing, Third generation sequencing, Gut content analysis, Community assembly

## Abstract

Large-scale studies on community ecology are highly desirable but often difficult to accomplish due to the considerable investment of time, labor and, money required to characterize richness, abundance, relatedness, and interactions. Nonetheless, such large-scale perspectives are necessary for understanding the composition, dynamics, and resilience of biological communities. Small invertebrates play a central role in ecosystems, occupying critical positions in the food web and performing a broad variety of ecological functions. However, it has been particularly difficult to adequately characterize communities of these animals because of their exceptionally high diversity and abundance. Spiders in particular fulfill key roles as both predator and prey in terrestrial food webs and are hence an important focus of ecological studies. In recent years, large-scale community analyses have benefitted tremendously from advances in DNA barcoding technology. High-throughput sequencing (HTS), particularly DNA metabarcoding, enables community-wide analyses of diversity and interactions at unprecedented scales and at a fraction of the cost that was previously possible. Here, we review the current state of the application of these technologies to the analysis of spider communities. We discuss amplicon-based DNA barcoding and metabarcoding for the analysis of community diversity and molecular gut content analysis for assessing predator-prey relationships. We also highlight applications of the third generation sequencing technology for long read and portable DNA barcoding. We then address the development of theoretical frameworks for community-level studies, and finally highlight critical gaps and future directions for DNA analysis of spider communities.

**Electronic supplementary material:**

The online version of this article (10.1007/s00427-020-00652-x) contains supplementary material, which is available to authorized users.

## Introduction

Ecological communities are defined by both the organisms that persist within habitats, and the interactions that shape the assembly and diversity patterns of these organisms. Historically, characterizations of abundance, richness, relatedness, and interactions across entire communities have been limited to taxa that are readily identifiable or have been done on a sufficiently small scale that the laborious process of quantifying all community members and their interactions has been feasible (Gruner 2004; Krushelnycky et al. 2007). Predator-prey interactions have largely been based on observation (Binford 2001; Hiruki et al. 1999), detailed morphological examination of gut contents (Grey et al. 2002; Lafferty and Page 1997), or the analysis of stable isotope data (Wise et al. 2006). The recent advent of molecular metabarcoding approaches is just starting to revolutionize our ability to characterize biological communities (Cristescu 2014). In particular, data on small invertebrates, which make up the foundation of food webs and play central roles in ecosystem function, can be obtained on larger scales and in greater detail than ever before. We use spiders, some of the most phylogenetically and ecologically diverse predators on Earth (Foelix 2011), to illustrate the potential of such approaches for understanding community assembly.

In the last two decades, DNA barcoding, the sequencing of short species-specific amplicons, has considerably simplified community analyses (Hebert et al. 2003). DNA barcodes can provide information on genetic variation within and between species, rapidly assign taxonomic status across divergent lineages (Hebert and Gregory 2005), and identify the prey composition of predators’ gut contents (Agustí et al. 2003; Greenstone and Shufran 2003). However, traditional Sanger sequencing-based DNA barcoding protocols can be prohibitively expensive and laborious when large community samples have to be processed. The emergence of high-throughput sequencing technologies (HTS) has been a significant step forward in recent years, greatly reducing both the cost and the labor required for biodiversity studies (Bohmann et al. 2014; Taberlet et al. 2012). These technologies enable simultaneous processing of DNA barcodes for thousands of specimens (Shokralla et al. 2015; Srivathsan et al. 2019; Meier et al. 2016) with considerably improved phylogenetic resolution (Krehenwinkel et al. 2019a). Metabarcoding makes it possible to characterize the species composition of whole communities (Cristescu 2014; Yu et al. 2012) and identify the makeup of the predators’ diets in an unprecedented detail (Piñol et al. 2014; Verschut et al. 2019). Recent developments even enable mobile DNA barcoding under remote field conditions (Menegon et al. 2017; Pomerantz et al. 2018).

Here, we provide an overview of available HTS-based methods, focusing specifically on the use of genetic and genomic data for characterizing community structure and function in spiders. Within this context, we first discuss DNA barcoding for taxonomic and phylogenetic assignments, metabarcoding for community analysis, recent developments in long-read sequencing technology, and portable field barcoding solutions. We then review the application of DNA barcoding for gut content analysis to assess predator-prey associations. We additionally discuss the development of theoretical models to apply to the DNA-based community analyses. Finally, we address promising avenues of future research.

## DNA barcoding and metabarcoding for community analysis

### DNA barcoding in spiders: An overview

As major predators of invertebrates, spiders are a central element of terrestrial food webs and perform key roles in community function and assembly (Nyffeler and Birkhofer 2017). They provide important ecosystem services, such as pest control (Riechert and Lockley 1984; Thomson and Hoffmann 2010) and at the same time make up much of the diet of higher order predators such as birds (Nyffeler et al. 2018). Most habitats harbor diverse communities of spiders with complex ecological interrelationships (Kennedy et al. 2019; Raso et al. 2014). Consequently, the diversity of spiders and their manifold interactions with other species must be understood in order to characterize community assembly in terrestrial ecosystems. Spider communities are usually composed of ecologically and morphologically distinct taxa, stemming from deeply divergent evolutionary lineages (Cardoso et al. 2011). The identification of different groups often requires specialized taxonomic expertise, a skillset which is rapidly disappearing (Agnarsson and Kuntner 2007).

**Fig. 1:**
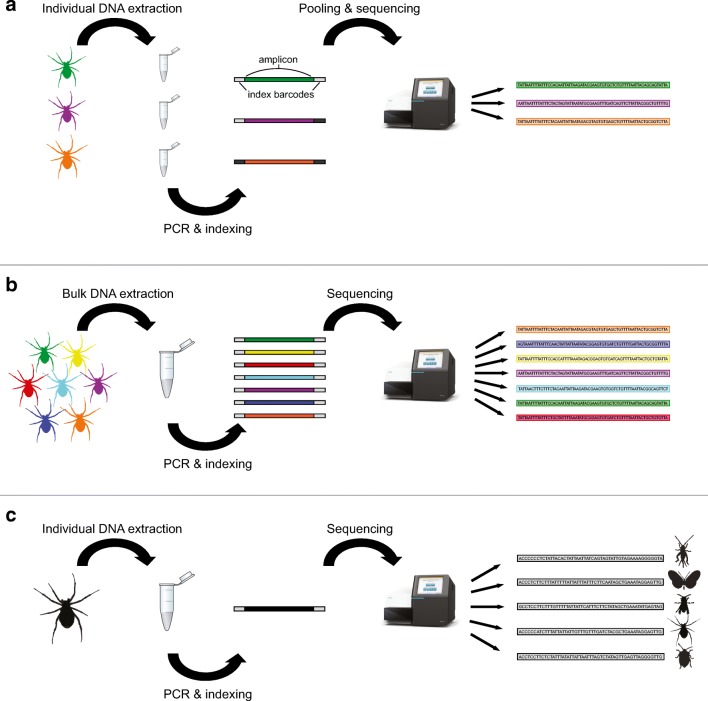
Summary of applications of Illumina amplicon sequencing for DNA barcoding and metabarcoding of spiders. A) In individual DNA barcoding, DNA from each specimen is extracted, then the desired fragment is amplified in a PCR and tagged with a unique combination of index barcodes before all samples are pooled and sequenced. B) In bulk metabarcoding, DNA extraction is performed on pools of multiple specimens. This greatly reduces the number of PCRs and index combinations needed per specimen. C) For molecular gut content analysis, DNA is extracted from individual predator specimens, and PCR primers are chosen to amplify prey taxa while minimizing amplification of the predator itself

The task of characterizing the entire spider communities has been greatly simplified by DNA barcoding (Barrett and Hebert 2005; Čandek and Kuntner 2015; Crespo et al. 2018; Fig. 1). DNA barcoding of spiders is usually based on the 650-bp “barcode region” of the mitochondrial COI gene, which provides good taxonomic resolution in this group (Čandek and Kuntner 2015). As part of the mitochondrial genome, COI is maternally inherited and not affected by recombination. Mitochondria occur in most tissues in high copy numbers and are thus easily accessible for PCR amplification, even in degraded samples. The gene also evolves relatively quickly, making it suitable to distinguish even recently diverged species and recover intraspecific variation (Hajibabaei et al. 2007).

DNA barcodes garnered much enthusiasm after their initial establishment, with some authors even suggesting that traditional taxonomic methods be entirely replaced with a DNA sequence divergence-based taxonomy (Meierotto et al. 2019; Tautz et al. 2003). However, a divergent barcode sequence recovered from an unknown specimen is not enough to indicate the species status (Moritz and Cicero 2004; Obertegger et al. 2018). Instead, DNA barcodes can serve as a valuable complement to traditional taxonomy by facilitating the identification of divergent lineages, including cryptic species (Wang et al. 2018). This has proven to be useful in some spiders with ambiguous morphological differentiation, which show sufficiently deep genetic divergence to be considered separate species (Leavitt et al. 2015; Starrett and Hedin 2007; Crespo et al. 2018).

Multiple primers with various levels of taxonomic specificity are available for DNA barcoding of spiders (Blagoev et al. 2016; Krehenwinkel et al. 2018; Supplementary Table 1). The resulting barcode sequences can be compared to reference databases to assign specimens to species (Barrett and Hebert 2005; Robinson et al. 2009). Alternatively, the presence of a so-called DNA barcode gap between interspecific and intraspecific genetic divergences in the COI gene (Hebert et al. 2004a, b) can be used to identify putative species from the DNA barcode data. There is no universal rule for genetic distances to warrant “species” status; instead, the barcode gap must be evaluated on a lineage-specific basis. This approach was demonstrated to work well in orb-weaver and wolf spiders (Čandek and Kuntner 2015). Automated approaches aiding in the discovery of barcode gaps and resulting species are available (Puillandre et al. 2012). Yet another approach is the grouping of barcodes from a community into clusters of similarity, the so-called operational taxonomic units (OTUs) (Edgar 2013). These clusters, usually based on a maximum sequence divergence of 3%, are then treated as biological entities. Even though OTU clusters do not necessarily correspond to actual species, this approach can be very useful when reference libraries are incomplete (Dopheide et al. 2019) and large numbers of sequences need to be processed. Clustering approaches can also be phylogenetically informed, resulting in more accurate approximations of real species (Fujita et al. 2012; Zhang et al. 2013).

### Problems with DNA barcoding, and approaches to mitigate problems

A significant obstacle to DNA barcoding is the incompleteness of the barcode reference libraries. Because many species have not yet been added to these libraries, often specimens can only be identified to a relatively coarse taxonomic level such as order or family. Even though substantial contributions to reference databases have been made in recent years (Astrin et al. 2016; Blagoev et al. 2016), given the sheer taxonomic diversity of spiders, a large proportion of species is still not represented. There are two major bottlenecks in the generation of barcode reference libraries. First, the identification of species is time-consuming and requires taxonomic expertise, which may not be available (Agnarsson and Kuntner 2007). Misidentifications or sequencing of contaminant DNA can then lead to erroneously assigned barcode sequences in the database. Second, many species are rare or difficult to collect, and thus represented by little more than type material. Museum collections are therefore an indispensable resource for DNA barcoding. In spiders, this is particularly feasible because the standard storage medium for spiders – ethanol – is an effective DNA preservative. Only slight modifications of DNA extraction and PCR protocols are needed to recover the reduced and fragmented DNA from historical specimens (Krehenwinkel and Pekár 2015; Miller et al. 2013). Several primer combinations are available to target short, so-called mini-barcodes, which are suitable for amplification of older specimens (Supplementary Table 1). In spiders, barcode analysis of historical specimens has provided valuable insights into the taxonomic assignment of species (Cotoras et al. 2017) and historical changes in genetic variation (Krehenwinkel and Tautz 2013).

Another challenge for DNA barcoding is that short-mitochondrial amplicons such as COI can yield biased biodiversity assessments when used in isolation (Krehenwinkel et al. 2018). Mitochondrial divergence patterns do not necessarily parallel species divergence but are influenced by numerous different factors. For example, male-biased gene flow can lead to highly divergent mitochondrial genomes in the absence of nuclear differentiation (Krehenwinkel et al. 2016). Conversely, introgression can result in complete homogenization of the mitochondrial gene pools, despite divergent nuclear genomes (Irwin et al. 2009). Infections with endosymbiotic bacteria can mimic various demographic scenarios of over- and under-differentiation of mitochondrial genomes compared to the nuclear background (Hurst and Jiggins 2005). Moreover, nuclear mitochondrial pseudogenes (NUMTs) can be recovered as barcode sequences, leading to incorrect taxonomic assignments and biased phylogenetic inferences (Bensasson et al. 2001). To avoid these pitfalls, it is often recommended to use multiple loci for DNA barcoding (Dupuis et al. 2012). Information from the unlinked loci in the nuclear genome is particularly important and can aid with DNA barcode-assisted taxonomic discoveries, e.g., for testing hypotheses on cryptic species (Satler et al. 2013). Although many popular nuclear markers evolve much more slowly than COI, they still show comparable patterns of genetic divergence when intraspecific and interspecific divergence rates are compared (Supplementary Fig. 1). Multilocus data can also increase the phylogenetic resolution of DNA barcoding, which is very limited when analyses are based on a single mitochondrial amplicon (Krehenwinkel et al. 2018).

### High throughput sequencing-based DNA barcoding

DNA barcoding is traditionally based on Sanger sequencing, requiring separate sequencing reactions for every sample. With a total cost of $ 5–10 per sequence, this method can be prohibitively expensive for community-level studies. A cost-efficient alternative is high-throughput amplicon sequencing (Kozich et al. 2013). Illumina technology, for example the MiSeq, with its maximum read length of 2 × 300 bp, is highly suitable for DNA barcode generation (Shokralla et al. 2015). Due to limitations in read length, HTS-based DNA barcoding usually relies on shorter amplicons than the complete 650 bp barcode (Leray et al. 2013). Alternatively, the complete barcode can be recovered by sequencing multiple overlapping amplicons.

**Fig. 2:**
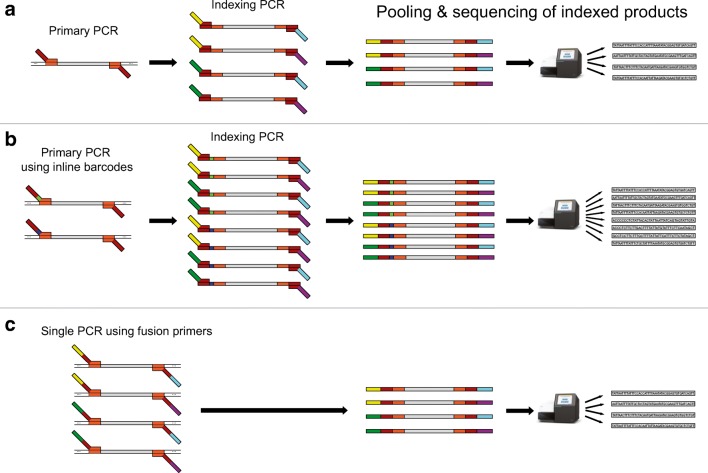
Dual indexing strategies for Illumina sequencing. A) Library preparation can be accomplished in two separate PCRs. In the first PCR, the DNA barcode specific primers contain added tails (in brown). Second PCR primers then bind to those tails and incorporate unique barcode identifiers as well as sequencing adapters to each sample. A unique barcode combination is used for each sample. B) Throughput can be increased with the use of inline barcodes (light green and indigo) attached to the 5′-end of the first-round PCR primer. They multiply the number of unique barcode combinations available. Here, we show inline barcodes only on the forward primer, but they can also be incorporated into the reverse primer. C) Fusion primers can be used so that only one round of PCR is necessary. The desired fragment is amplified and indexed simultaneously

Illumina amplicon sequencing is distinguished by a very simple library preparation process. Most commonly, a two-step PCR is used, in which the target sequence is amplified in the first round of PCR (Fig. 2). Dual indexes for unique sample tagging and the necessary adapters for sequencing are then incorporated in the second round of PCR (Lange et al. 2014). This approach accommodates thousands of samples in a single sequencing run. Multiplex PCRs targeting multiple unlinked loci can additionally reduce the necessary number of PCRs (Krehenwinkel et al. 2018; Macías-Hernández et al. 2018), and inline barcodes attached to the first-round PCR primers allow for a further increase in sample number (Sternes et al. 2017). Alternatively, fusion primers including sample tags and sequencing adapters can be used. This allows library preparation to be accomplished in a single PCR (Kozich et al. 2013; Fadrosh et al. 2014) but limits the flexibility to target multiple amplicons. Further reductions in processing cost can be achieved by limiting the number of DNA extractions. This can be done by pooling specimens of divergent lineages, then performing bulk extractions (de Kerdrel et al. 2020). PCR and library preparation are then performed on the pooled extract, and the resultant sequences are assigned back to their specimens using a reference database. One other option is to omit DNA extraction entirely and instead use direct PCR (Wong et al. 2014). Here, specimens are dropped directly into the PCR buffer, and the traces of DNA they release are sufficient for barcode amplification. This method has been tested and established in different insect groups (Thongjued et al. 2019; Yeo et al. 2018) but has yet to be optimized for spiders.

By limiting the number of DNA extractions, using multiplex PCRs, and using multiple levels of sample indexing, barcodes can now be generated at a cost of $ 0.2–1 each and with a considerable reduction of workload (de Kerdrel et al. 2020; Meier et al. 2016; Srivathsan et al. 2019). This enables researchers to generate barcode sequences for thousands of specimens, thereby allowing estimates of both abundance and taxonomic richness within a community (see below). Recent reductions in cost have even led to the suggestion of a reverse DNA barcoding workflow, in which all specimens in a collection are barcoded and only divergent lineages are selected for further morphological analysis (Wang et al. 2018).

Using multiplex PCR approaches, sequences for multiple independent loci can be generated in parallel, greatly improving the phylogenetic resolution of the generated data. Knowing the evolutionary relationships among taxa in a community is critical for understanding the processes underlying community assembly (Barker 2002). Currently, phylogenetic analyses often rely on information from hundreds or thousands of loci, for example, inferred from whole transcriptome sequencing (Foley et al. 2019) or the targeted enrichment of ultra-conserved elements (Kulkarni et al. 2020). While such data offer unprecedented phylogenetic resolution, their generation is expensive and laborious. These methods are thus not feasible for phylogenetic analyses at the community level, where information for thousands of specimens has to be generated in parallel. This makes multiplexed amplicon sequencing an attractive alternative to phylogenomic approaches for community phylogenetic analyses.

### Community metabarcoding

Reductions in cost and processing effort have made DNA barcoding suitable even for the analysis of large community samples. However, processing all specimens individually still amounts to a considerable workload and cost. Metabarcoding offers a simple alternative to a single-specimen DNA barcoding and is therefore quickly increasing in popularity (Gibson et al. 2014; Yu et al. 2012). In metabarcoding, bulk samples are extracted and DNA barcode sequences generated for the pooled community. Based on the sequence similarity, the recovered barcodes are then clustered into OTUs. Community diversity is estimated based on the number of recovered OTU sequences. In order to achieve a comprehensive taxon recovery in metabarcoding experiments, the use of more than one amplicon is advisable (Krehenwinkel et al. 2018; Zhang et al. 2018). Recent developments in clustering algorithms (Edgar 2018) also allow the inference of haplotypic information from bulk metabarcoding data. This way, even the intraspecific genetic variation can be estimated within whole biological communities (Elbrecht et al. 2018).

Due to its speed, accuracy, and cost efficiency, metabarcoding is now often the method of choice for arthropod community analysis. A major drawback of this approach, however, is that it only yields a list of OTU sequences, which cannot be linked back to individual specimens because the DNA is bulk-extracted from mixed samples. As sequences cannot be assigned back to specimens, individual sequences from multilocus barcoding cannot be linked together, limiting the phylogenetic resolution of this approach unless the specimens can be linked to an extensive reference library (de Kerdrel et al. 2020). Metabarcoding can also lead to inflated diversity estimates, as spurious sequences coamplify with the targeted specimen’s DNA barcodes. These include NUMTs, non-target species such as parasitic fungi or nematodes associated with the specimen, and chimeras resulting from linking of incomplete PCR products of different taxa (Elbrecht et al. 2017). Chimeras can be removed efficiently with appropriate software solutions (Edgar 2013) and non-target taxa by comparing the resulting data against a reference database. However, the removal of NUMTs is more challenging and has not been fully resolved, especially when NUMTs retain an intact reading frame.

Another issue with metabarcoding is that many current protocols are performed destructively, i.e., specimens are crushed in order to maximize the amount of DNA released for extraction. This inevitably leads to the loss of morphological information. This issue, however, can be circumvented by subsampling tissue from specimens before extraction. In spiders, it is common to extract DNA from one or more legs while leaving the rest of the specimen intact (Gillespie et al. 2018; Krehenwinkel et al. 2018). In addition, several nondestructive protocols have been developed to isolate DNA from specimens without compromising their morphological integrity, e.g., via a brief soak in lysis buffer (Andersen and Mills 2012; Porco et al. 2010). DNA may even be extracted directly from the collection medium (e.g., ethanol), because specimens leave trace amounts of DNA in the medium (Hajibabaei et al. 2012; Martins et al. 2019). However, the DNA recovered from nondestructive extraction of community samples may be biased toward those taxonomic groups that release DNA more readily than others, e.g., soft-bodied animals (Carew et al. 2018; Marquina et al. 2019).

### Quantitative metabarcoding and PCR-free metagenomics

While metabarcoding has been able to provide a highly accurate overview of a community’s species composition, it has not been possible to obtain accurate measures of abundance using this approach. This is because varying PCR efficiencies between different taxa inevitably lead to biased recovery of species abundances, sometimes by several orders of magnitude (Elbrecht and Leese 2015). Besides simple primer-template mismatches (Piñol et al. 2015), the GC content of the template and even the polymerase type can bias taxon recovery (Nichols et al. 2018). The effects of these biases are condition dependent and difficult to quantify. Even the effect of primer-template mismatches does not simply accumulate with mismatch number. Instead, position and type of mismatch can have widely different effects on amplification efficiency (Kwok et al. 1990). Nevertheless, the accurate quantification of relative abundances of taxa is critical for many biodiversity analyses, and much effort is therefore being made to optimize methods for quantitative metabarcoding (Krehenwinkel et al. 2017a; Piñol et al. 2019; Saitoh et al. 2016).

To overcome the amplification biases of metabarcoding, PCR-free approaches have been suggested (Jones et al. 2015). The simplest is shotgun sequencing of bulk samples, after which the generated reads are processed and compared against a reference database, an approach called metagenomics. However, this may not work well in spiders because very few spider genomes (nine, representing seven families) are currently available (Supplementary Table 2), making it difficult to identify the majority of sequences. A refinement of this method is “genome skimming,” in which mitochondrial sequences are filtered from the recovered reads after shotgun sequencing (Papadopoulou et al. 2015). The filtered reads can then be assembled into longer contigs sometimes even spanning the whole mitochondrial genome, allowing community analysis with considerable phylogenetic support (Crampton-Platt et al. 2016). However, although mitochondria are abundant in cells, mitochondrial DNA sequences usually do not exceed 1% of the read population of genomic libraries (Zhou et al. 2013). Genome skimming thus requires a very high sequencing coverage. Capture assays are another option: DNA barcode probes are used to capture barcode sequences from a community sample, allowing sequencing without PCR amplification (Shokralla et al. 2016). However, hybridization bias due to probe-target dissimilarities may also result in skewed abundance estimates; furthermore, a capture approach adds considerable cost and workload.

**Fig. 3:**
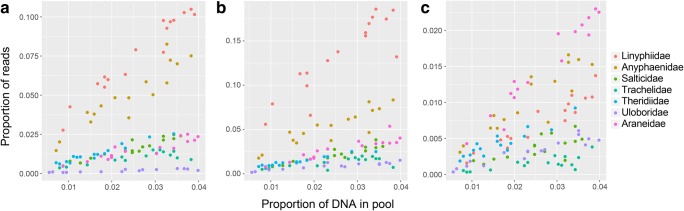
Association of the relative abundance of spider species from seven different families in mock communities of 46 different arthropod species, with the relative read count recovered for the species after sequencing. The plots show the association for A) nuclear 18SrDNA, B) nuclear 28SrDNA and C) mitochondrial COI. The abundance of the species in the respective communities is generally well correlated to the recovered read abundances. However, depending on marker and species, the slope of the association is very variable, such that accurate abundance estimates from read data would require careful calculation. Based on data from Krehenwinkel et al. 2017a

PCR-based metabarcoding is therefore still the most cost-effective and simple method for community analysis of spiders. Although PCR amplification bias can theoretically lead to skewed taxon abundances, this bias can be considerably mitigated using optimized protocols. Amplification with degenerate primers, or with primer binding in conserved DNA stretches, greatly improves taxon recovery while decreasing amplification bias (Krehenwinkel et al. 2017a). Furthermore, the response of individual taxa during a PCR is predictable, i.e., the relative abundance of a taxon in a community is linearly correlated with the recovered read abundance (Fig. 3). Only the slope of this correlation differs among taxa. If the slopes are known, then correction factors can be applied to estimate the relative abundance of taxa in a community (Thomas et al. 2016). The downside is that correction factors must be developed individually for different taxa.

### Environmental DNA metabarcoding of spiders

A popular application of metabarcoding is the analysis of environmental DNA (eDNA). Every organism leaves traces of DNA in the environment, for example, from feces, skin fragments, or saliva. These traces can be enriched, amplified, and sequenced, allowing characterization of whole communities without needing to collect the organisms. Much work on eDNA has focused on aquatic ecosystems, using DNA extracts from filtered water (Valentini et al. 2016). However, terrestrial organisms can also be detected using eDNA, for example from soil DNA extractions. Arthropods were recently shown to leave eDNA traces on wildflowers (Thomsen and Sigsgaard 2019). Thus, by washing eDNA off of plants, it may be possible to reconstruct associated arthropod communities.

### Third generation sequencing-based barcoding and metabarcoding

DNA barcoding and metabarcoding applications are currently limited by the relatively short read length of second generation HTS applications, which cannot recover the whole 650-bp COI barcode region as a single sequence (Piper et al. 2019). A solution to this limitation is provided by the third generation sequencing technologies. Oxford Nanopore Technologies (ONT) and Pacific Biosciences (PacBio) offer sequencing platforms that achieve read lengths superior to any previous sequencing technology, with reads of close to 2 megabases for ONT’s MinION platform (Payne et al. 2018). Both technologies are well suited for amplicon sequencing, and dual indexes can be easily incorporated during PCR, allowing processing of thousands of DNA barcodes in a single sequencing run. The PacBio Sequel (Hebert et al. 2018) and ONT’s MinION (Srivathsan et al. 2019) were recently suggested as cost-efficient alternatives to Sanger sequencing for the generation of DNA barcodes. ONT and PacBio platforms have also recently been used to sequence near complete nuclear ribosomal DNA clusters (Krehenwinkel et al. 2019a; Tedersoo et al. 2018). The advantage of rDNA barcoding is that conserved gene regions of the rDNA cluster can be used to design universal primers (anchored in the highly conserved 18S and 28S rDNA), which can resolve very old divergences (Hillis and Dixon 1991), while at the same time fast-evolving internal transcribed spacers (ITS) can be used to resolve relationships of closely related taxa (Schoch et al. 2012). Our work on spiders suggests that such long rDNA amplicons are a well-suited complement to COI-based DNA barcoding (Supplementary Fig. 2; Krehenwinkel et al. 2019a). Long rDNA amplicons also offer very good phylogenetic support and thus may constitute a cost-effective alternative to the multiplexed Illumina amplicon sequencing for the community-wide phylogenetic analysis.

The third generation sequencing platforms offer an unprecedented read length, but their major downside is a high raw read error rate. At about 5–30% (Tedersoo et al. 2018; Wick et al. 2018), ONT and PacBio sequencers’ raw read error rate is much higher than that of Illumina (error rate: 0.1–1%; Manley et al. 2016) and Sanger sequencing (error rate: 0.001–1%; Noguchi et al. 2006). However, highly accurate consensus sequences can be generated from ONT and PacBio data even at low coverage (Krehenwinkel et al. 2019a; Pomerantz et al. 2018). Recent advances show promise for further minimizing error. PacBio HiFi sequencing mode produces highly accurate reads using their circular consensus sequencing (CCS) technology, reducing raw read error to < 1% (Wenger et al. 2019). Similarly, rolling circle amplification can be used in metabarcoding applications to mitigate error rates of the nanopore-based sequencing platforms (Calus et al. 2018).

The possibility of long-read metabarcoding was also recently explored (Callahan et al. 2019; Krehenwinkel et al. 2019a; Tedersoo and Anslan 2019). Barcode sequences of several thousand base pairs for a whole community would greatly improve the phylogenetic resolution of metabarcoding and allow community-level phylogenetic analysis. However, the high raw read error rate poses a significant obstacle for accurate community characterization, as it is hard to distinguish whether a rare sequence variant belongs to a separate species in the community or is simply caused by sequencing error. Nonetheless, advances such as CCS and rolling circle amplification may soon solve this problem. One other issue is that community compositions of rDNA metabarcoding studies can be highly skewed, likely due to favorable PCR amplification of shorter rDNA fragments (Krehenwinkel et al. 2019a). Although the length of the nuclear rDNA region is relatively stable within spiders, biases can occur when additional taxa are included (Krehenwinkel et al. 2019a).

### Mobile DNA barcoding by third generation sequencing

A particular strength of ONT’s MinION is its portability. With the size of a USB stick, the device can be run outside of conventional laboratories (e.g., Menegon et al. 2017; Pomerantz et al. 2018). Using a mobile laboratory of miniaturized equipment, all steps from DNA extraction to PCR, library preparation and sequencing can be performed in the field using the MinION (Pomerantz et al. 2018; reviewed in Krehenwinkel et al. 2019b). While field-based DNA barcoding is an exciting perspective, it is unlikely to become the method of choice for community barcoding. Researchers usually have access to molecular laboratories that allow for more standardized and higher throughput sample processing than field-based assays. Yet, a minimalistic and mobile DNA barcoding system can be of great advantage when field sites are remote or hard to access, or when time is of the essence for swift generation of biodiversity information (reviewed in Krehenwinkel et al. 2019b). Examples include monitoring of disease outbreaks (Quick et al. 2016; Walter et al. 2017) or documenting the immediate effects of ecological disasters such as forest fires or pipeline spillages. Another advantage of mobile barcoding is that it allows for in situ species monitoring without having to remove organisms from their habitat or send samples internationally (Pomerantz et al. 2018). This is especially relevant for endangered species. In the case of spiders, non-lethal sampling protocols could be applied for site-based monitoring without directly affecting the population (Longhorn et al. 2007; Petersen et al. 2007).

## Trophic niche analysis by DNA barcoding

### DNA barcoding for gut content analysis: toward community-level food webs

Surprisingly little is known about the dietary ecology of spiders. While most spiders have long been understood as generalist predators, recent work also highlights many examples of dietary specialists, like termite feeders (Petráková et al. 2015), araneophages (Benavides et al. 2017; Wood et al. 2012) and even herbivorous species (Meehan et al. 2009; Nyffeler et al. 2016). The compilation of dietary information for spiders from observational data is very time-consuming and often inaccurate. The sheer diversity of spiders additionally complicates the task. More detailed dietary information is available chiefly for species being considered as potential biocontrol agents (Schmidt et al. 2014; Roubinet et al. 2017).

Molecular gut content analysis has simplified the task of characterizing spider prey communities and associated strengths of predator-prey interactions, thereby allowing a more accurate reconstruction of the often-cryptic arthropod food web (Sint et al. 2019). In the simplest case, spiders can be tested for consumption of specific prey taxa by subjecting a spider’s gut content to PCR assays using prey-specific primers (Schmidt et al. 2014; Whitney et al. 2018). This can be useful for determining whether a spider could serve as a biocontrol agent against a particular pest species. However, the limitation of this method is that the expected prey taxa must be known a priori, and specific PCR assays must be developed for every prey taxon. Multiplex PCR approaches (King et al. 2011; Roubinet et al. 2017) can broaden taxonomic coverage of prey detection but are still limited in their taxonomic breadth.

**Fig. 4:**
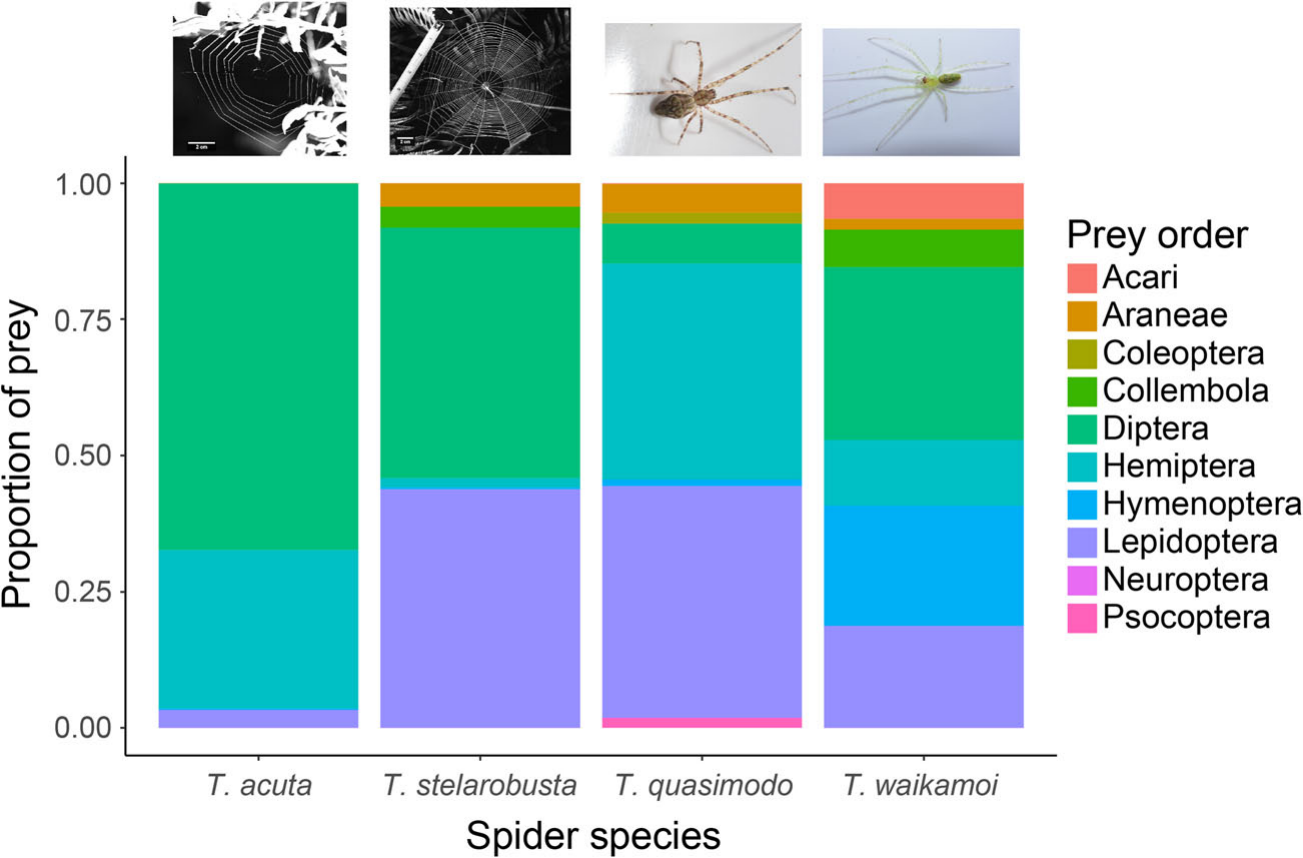
Order-level prey compositions for four sympatric *Tetragnatha* species from the Hawaiian island of Maui, as recovered by molecular gut content analysis based on a short COI amplicon. The different lifestyles of the web builders (*T. acuta* and *T. stelarobusta*) and free-hunting species (*T. quasimodo* and *T. waikamoi*) are reflected in divergent prey spectra. However, prey community differences also become apparent within web builders and free hunters. This effect may be due to interspecific differences in microhabitat and prey capture strategy. The green *T. waimamoi* resides and hunts on green leaves, while the brown *T. quasimodo* occurs on tree bark or dead leaves. The two web builders are distinguished by different web mesh widths, selectively catching different arthropod groups. Based on data from Kennedy et al. 2019

A more complete prey spectrum can be recovered via metabarcoding of the gut contents (Deagle et al. 2009; Leray et al. 2013). In principle, the same DNA barcoding approaches used for community characterization can be applied to gut content analysis, i.e., by treating the prey DNA inside the gut as a “community.” However, there are some additional considerations for tailoring these approaches to the specific conditions affecting DNA extracted from the gut. PCR inhibitors may coprecipitate with the DNA, requiring additional purification of DNA from gut extractions. Also, prey DNA from the predator’s guts is often degraded and present at much lower concentrations than the DNA of a single specimen in a bulk community extract. Hence, short PCR amplicons are usually targeted to achieve a complete prey spectrum (Zeale et al. 2011; Kamenova et al. 2018). Gut content metabarcoding has become increasingly popular and has recently provided numerous novel insights into the trophic ecology of spiders. Examples include trophic niche differentiation within an adaptive radiation (Fig. 4; Kennedy et al. 2019), the effect of grazers on prey communities (Schmidt et al. 2018), ontogenetic shifts in diet (Verschut et al. 2019), and a stable diet despite differences in available prey communities along an elevational gradient (Eitzinger et al. 2019). Improved resolution is often achieved by combining HTS-based gut content screening with stable isotope analysis (Hambäck et al. 2016; Kennedy et al. 2019).

### Enrichment of prey DNA

**Fig. 5:**
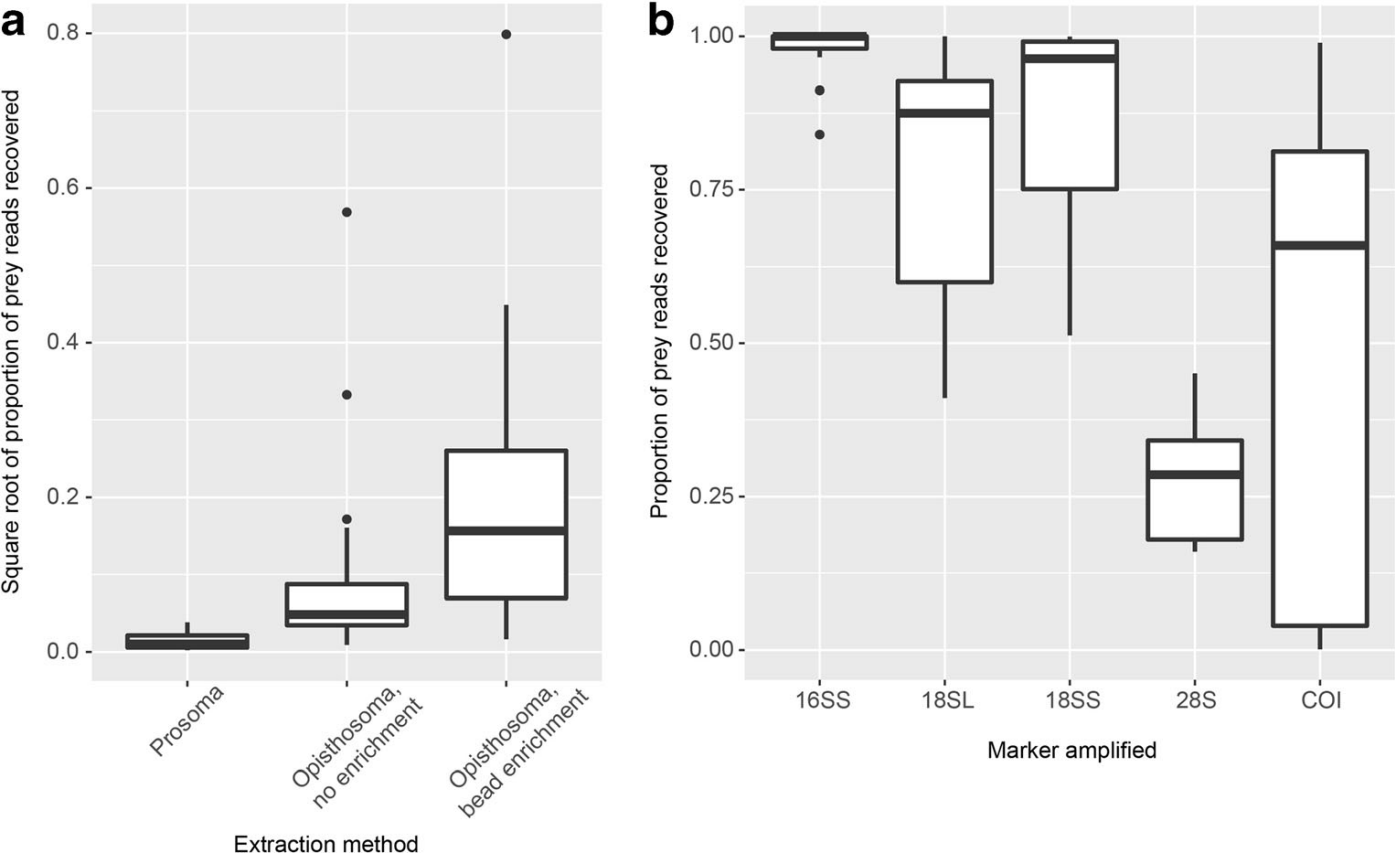
Enrichment of prey DNA from extractions of spiders. A) The recovered relative amount of prey DNA of *Hololena adnexa* in relation to spider DNA increases significantly when DNA extractions are performed from the opisthosoma rather than the prosoma. The yield can be further increased by using a bead protocol to enrich the low molecular weight DNA from the DNA extract. This works because prey DNA rapidly degrades in the spider’s digestive tract. B) Enrichment of prey DNA in 12 spider species from seven families by using different lineage-specific primers and in comparison to a commonly used COI primer pair. Based on spider-specific 3′-primer mismatches, the amplification of spiders can be considerably reduced, enriching the prey DNA during PCR. Based on data from Krehenwinkel et al. 2017b, 2019c

Dissecting the gut of a spider for prey recovery is time-consuming and laborious. A highly simplified approach was thus suggested by Piñol et al. (2014). In their study, the authors used DNA extractions from whole spider bodies and amplified DNA barcodes using universal primers. Predator barcodes, which co-amplified during PCR, were removed from the analysis, and the prey spectrum is reconstructed from the remaining sequences. As universal primers are needed in order to recover a full prey spectrum, a serious problem of this approach is that both predator and prey will be amplified. Consequently, the overabundant predator DNA can completely outcompete the prey DNA during PCR. Recent work suggests the use of predator-specific blocking primers, but due to the close relatedness of prey and predator, this approach has had only limited success in spiders (Michálek et al. 2017; Toju and Baba 2018). Another option is to enrich prey DNA from spider extracts. Prey DNA is quickly degraded in the spider’s digestive tract. By separating intact high molecular weight DNA from degraded DNA fragments, prey DNA can be significantly enriched (Fig. 5; Krehenwinkel et al. 2017b). However, this method will only work well if the predator DNA is not degraded; therefore, it is not suitable for old or poorly preserved specimens. An additional enrichment of prey DNA can be achieved by extracting DNA from the spider’s opisthosoma only, which contains the majority of the animal’s digestive tract (Krehenwinkel et al. 2017b; Macías-Hernández et al. 2018).

To further optimize prey recovery and reduce the necessary sequencing depth, lineage-specific PCR was recently suggested (Fig. 5; Krehenwinkel et al. 2019c). Single mismatches at the 3′-end of a PCR primer can lead to a massive drop in amplification efficiency (Kwok et al. 1990). If primers are designed in very conserved regions but end at a lineage-diagnostic SNP, which distinguishes spiders from their insect prey, spiders will be mostly blocked from amplification. At the same time, the primer still amplifies a wide variety of arthropod prey. Blocking spiders from amplification also enables detection of prey for very long time periods, possibly up to a month after feeding (Krehenwinkel et al. 2019c). A downside of this approach is that spider-spider predation cannot be detected. While prey enrichment can now be routinely performed from spider gut content, further standardizations of the protocol may be necessary to integrate the resulting data into previously generated prey community data.

### Non-lethal monitoring of spider prey communities

All methods mentioned above rely on DNA extraction from spiders or their body parts. Corse et al. (2019) suggest the use of DNA extracts from spider webs as an alternative source of prey DNA, without harming the spider. This method has several drawbacks. First, spider webs can collect airborne DNA from the environment, in addition to “bycatch” of insects that the spiders do not eat. This can lead to false positives if DNA from webs is used as a proxy for the spider’s diet. Also, many spiders rebuild their webs on a daily basis. Web DNA then only allows detection of the daily prey catch (as well as the bycatch described above), in contrast to several weeks recovered by gut content analysis. Many spiders do not spin capture webs but are active hunters, additionally limiting the broad applicability of this method. Another alternative was suggested by Sint et al. (2015), who used DNA extracted from spider feces as source of prey DNA. This is a promising approach but may be logistically challenging, as spiders must be kept in captivity until feces can be collected. Moreover, recent work in carabid beetles has shown that feces recover a less diverse, and therefore biased, prey spectrum compared to gut content extractions (Kamenova et al. 2018).

### Pitfalls of HTS-based gut content analysis and how to avoid them

HTS-based gut content analysis has been shown to yield reliable and comprehensive prey spectra, allowing exploration of food web structure in whole communities of spiders. However, the method still has several issues. PCR-based amplification of prey DNA is very sensitive to contamination. Theoretically, a few molecules of insect DNA are sufficient to be amplified. This DNA can also derive from external sources, e.g., from insects caught or stored together with the spider specimen. This contamination can be minimized by bleach treatment of the collected specimens before gut content analysis (Greenstone et al. 2012). Another source of contamination is parasitoid larvae, which may be located inside the spider but are not actual prey. Additional organisms inside the spider’s tissue, such as nematodes, fungi, and bacteria, can also be coamplified along with the prey. Prey data must therefore be carefully analyzed to identify such potentially confounding factors.

Amplification bias can also strongly affect taxon recovery in HTS-based gut content analysis. This problem can be mitigated using multiplex PCR assays targeting several loci (Krehenwinkel et al. 2019c), which enables a good qualitative recovery of prey. Accurate quantitative assessments of prey communities, however, are not possible. This is partly due to biased amplification of different taxa, and partly to the timing of prey consumption. DNA of the recently ingested prey will outweigh that of earlier meals, which will already be mostly degraded. However, by completely omitting quantitative information, the prey spectrum is artificially biased toward very rare taxa; thus, the number of reads obtained for different prey taxa should be taken into account even if abundance per se cannot be reliably inferred (Deagle et al. 2019).

Another shortcoming of current HTS-based protocols is that they fail to detect cannibalism. The short amplified barcode sequences for gut content analysis are usually shared within a species, making it impossible to distinguish the DNA of cannibalized prey from that of the predator. Recent work (Michálek et al. 2017) suggests using intraspecific haplotypic variation to identify cannibalism events, but this method only works if the spider population has very high-haplotypic variation. Hence, in most spider species, the number of cannibalism events would be considerably underestimated.

Secondary predation is yet another issue, particularly for protocols with a long detection half-life of prey DNA. For example, gut DNA extracts of a spider that has fed on a ladybird (Coccinellidae) may also contain DNA of the ladybird’s own aphid prey. However, secondary prey DNA is expected to be more degraded and less abundant than the DNA of an actual prey item, so a careful analysis of prey community data and the development of sequence coverage cutoffs may mitigate this issue. Such sequence coverage cutoffs will have to be derived experimentally in the future to enable accurate assignments of real prey taxa in gut content studies.

## Future outlook: practical and theoretical developments in the field

### Development of laboratory and field protocols

Recent technological developments have greatly contributed to the field of DNA barcoding. Whole communities can now be routinely characterized for manageable cost and effort. However, the field is still in its infancy, and further developments are warranted. One important focus is the completion of DNA barcode reference libraries. Only with complete reference databases can DNA barcoding be used to its full potential. Museum collections are a promising source of specimens from which to generate barcode data for the world’s spider biota. Also, future species descriptions should be coupled with the deposition of a DNA barcode sequence. Considering the limitations of the single-locus DNA barcoding, new unlinked barcode loci should additionally be developed. These should include information from the nuclear genome and be variable enough to distinguish species but also include conserved sequences which allow the design of universal primers. A set of multiple, unlinked DNA barcoding loci would greatly facilitate taxonomic discoveries in spiders and may aid in community phylogenetic analysis.

A focus of future research should also be on the optimization of quantitative taxon recovery from metabarcoding. Alternatively, further developments in amplification-free approaches may lead to a drop in cost, allowing this method to be applicable to the whole-community samples. Further optimizations should be performed on long read protocols so that they can be used for accurate metabarcoding analyses. This would considerably improve the phylogenetic resolution of metabarcoding data. With future simplifications to the protocol, portable barcoding could develop into a routine methodology for the exploration of remote ecosystems around the world. Gut content sequencing is currently revolutionizing our understanding of cryptic prey-predator interactions in arthropod communities. With further experimental developments, for example, into the avoidance of false positives and the enrichment of prey DNA, the methodology will enable an in-depth understanding of the arthropod food web structure, which is also critical for understanding the food web relationships at higher trophic levels.

### Linking theoretical biology and DNA barcoding

High throughput sequencing-based DNA barcoding and metabarcoding have provided scientists with community-level datasets of unprecedented completeness and resolution. Nevertheless, the theoretical tools available for analyzing these data are still somewhat limited. Recent efforts, however, show great promise for improving the power and accuracy of DNA barcode data for the analysis of community species richness, abundance, phylogenetics, and interactions. Here we provide an overview of the current developments and future perspectives of integrating DNA barcoding data into theory.

Theoreticians are facing new opportunities for making inferences about past processes that have contributed to structuring communities using community-scale sequence data. Events at different timescales are recorded in different aspects of these data, with abundance distributions reflecting short, ecological timescales, population genetic variation reflecting medium timescales, and phylogenetic diversity reflecting long timescales. For example, if abundances can be estimated from bulk-sampled sequence data using metabarcoding, then a variety of methods can be applied to differentiate neutral from non-neutral processes (Harpole and Tilman 2006; Tilman 2004), estimate assembly model parameters (Haegeman and Etienne 2017), or infer equilibrium state of the community using mechanistic theory (Jabot and Chave 2011) or tools from statistical mechanics (Harte and Newman 2014; Rominger and Merow 2017).

The distribution of genetic variation within a community provides another axis of information which is complementary to the abundance distribution (Vellend 2005; Vellend et al. 2014). Recently, Overcast et al. (2019) described a mechanistic model of community assembly that can generate linked patterns of abundance and genetic diversity under an assumption of joint ecological (Hubbell 2011) and evolutionary (Kimura 1983) neutrality to estimate community abundance structure using only intraspecific genetic variation. This method can serve as an alternative or a complement to estimates of abundance distributions from metabarcode data, which are confounded by PCR amplification bias as described above. As a proof of concept for this method, Overcast et al. (2019) analyzed the densely sampled abundances and community-scale population genetic data (COI sequences) from a community of spiders on La Réunion (Emerson et al. 2017) and demonstrated that the abundance structure of the community could be accurately estimated using only the intraspecific genetic variation.

Analysis of the community phylogenies provides a deep-time lens on community structure which can be used to estimate speciation and extinction rates (Manceau et al. 2015) and make inferences about diversification processes (Emerson and Gillespie 2008; Morlon 2014; Pearse et al. 2014). Recent methods have also been developed to simultaneously model trait evolution and species diversification (Weber et al. 2017) to investigate the importance of competition in shaping evolutionary radiations (Aristide and Morlon 2019), and the joint contribution of competition and environmental filtering in structuring ecological communities (Ruffley et al. 2019). Community-scale trait data can also be analyzed along with metabarcoding data in a hierarchical modeling framework to further account for feedbacks among processes happening at disparate timescales (Overcast et al. n.d.). Such theoretical developments enable increasingly reliable and detailed inferences on past processes in shaping present-day patterns, yielding many exciting new perspectives on community assembly.

## Electronic supplementary material

ESM 1 (DOCX 826 kb)
