# Supplementary material for: High-throughput sequencing for community analysis: the promise of DNA barcoding to uncover diversity, relatedness, abundances and interactions in spider communities
Source: Dev Genes Evol. 2020 Feb 10;230(2):185–201. doi: 10.1007/s00427-020-00652-x (PMC7127999; doi:10.1007/s00427-020-00652-x)
Supplement: Supplementary file 1 — (DOCX 826 kb) [file 427_2020_652_MOESM1_ESM.docx]

**High throughput sequencing for community analysis: The promise of DNA barcoding to uncover diversity, relatedness, abundances and interactions in spider communities**

Susan R. Kennedy^1^, Stefan Prost^2,3^, Isaac Overcast^4,5^, Andrew J. Rominger^6^, Rosemary G. Gillespie^7^, Henrik Krehenwinkel^8^*

^1^Okinawa Institute of Science and Technology, Biodiversity and Biocomplexity Unit, Onna, Okinawa, Japan; ORCID: 0000-0002-1616-3985

^2^LOEWE-Centre for Translational Biodiversity Genomics, Senckenberg Museum, Frankfurt, Germany; ORCID: 0000-0002-6229-3596

^3^South African National Biodiversity Institute, National Zoological Garden, Pretoria, South Africa

^4^Graduate Center of the City University New York, NY, USA; ORCID: 0000-0001-8614-6892

^5^Ecole Normale Supérieure, Paris, France

^6^Santa Fe Institute, Santa Fe, NM, USA; ORCID: 0000-0003-3755-4480

^7^University of California Berkeley, Environmental Sciences Policy and Management, Berkeley, CA, USA; ORCID: 0000-0003-0086-7424

^8^Trier University, Department of Biogeography, Trier, Germany; ORCID: 0000-0001-5069-8601

*Corresponding author: [krehenwinkel@uni-trier.de](mailto:krehenwinkel@uni-trier.de)

**Supplementary Information**


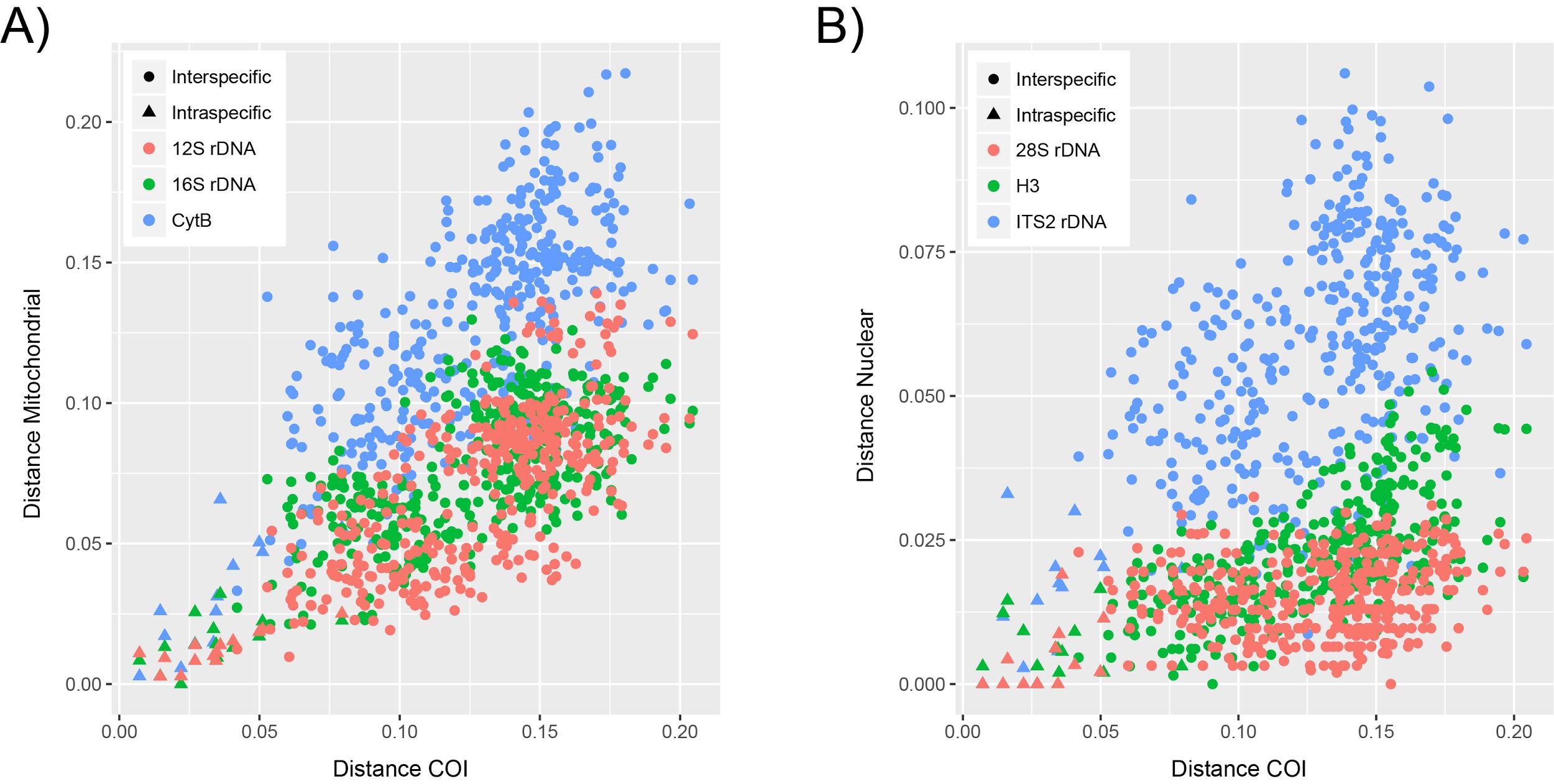


**Supplementary Fig. 1** Intraspecific and interspecific pairwise genetic distances among 29 endemic Hawaiian spider species of the genus *Tetragnatha*. The genetic distances for the commonly used DNA barcode marker COI are shown on the X-axis in comparison to A) other mitochondrial and B) nuclear markers suitable for spider taxonomy on the Y-axis. While all markers except for mitochondrial Cytochrome B generally show shorter genetic distances than COI, they still show comparable divergence patterns. Based on data from Krehenwinkel et al. 2018


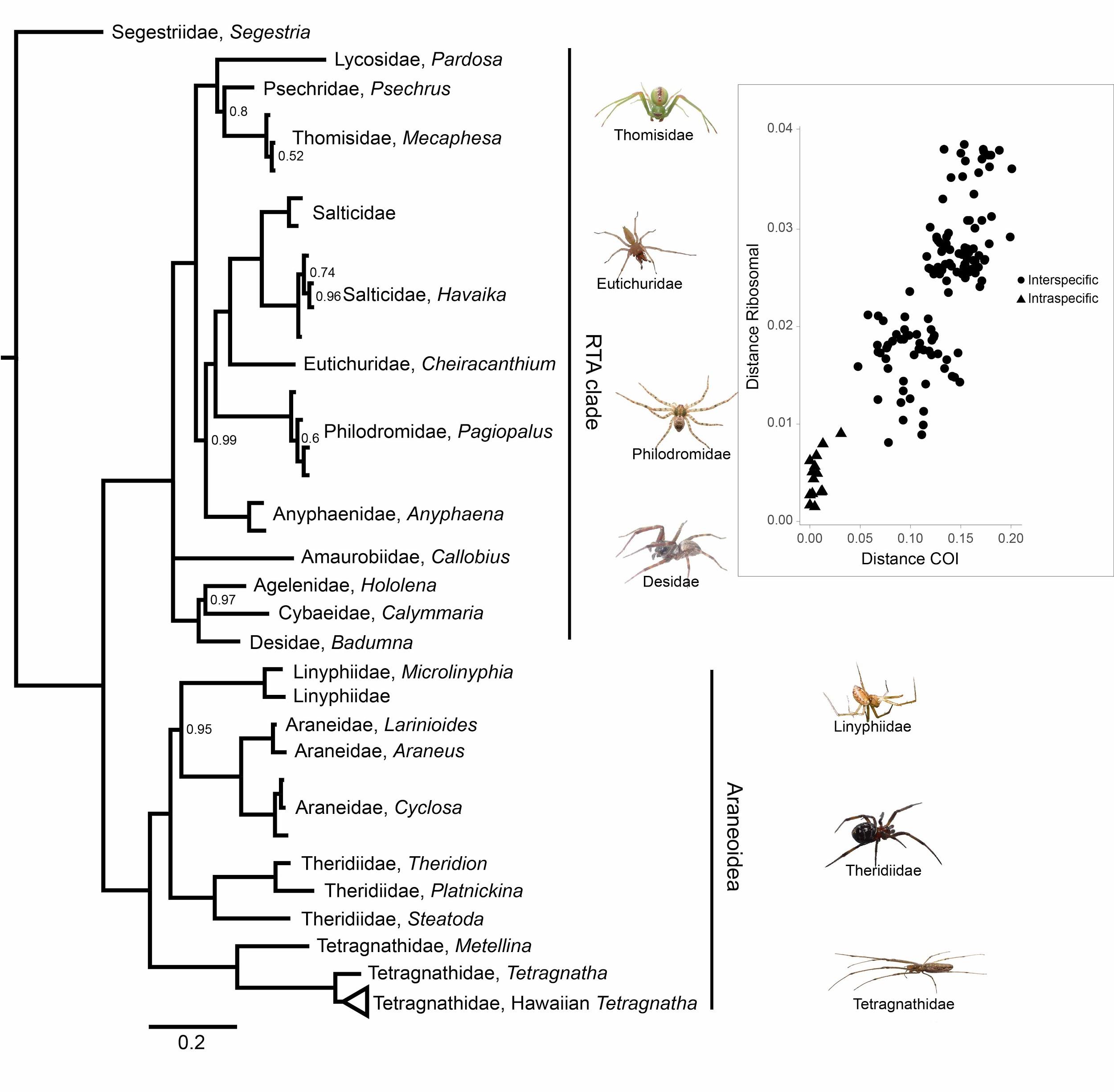


**Supplementary Fig. 2** Bayesian phylogeny for spiders from 16 families, based on a 4200-bp alignment of the nuclear ribosomal cluster, covering 18S, ITS1, 5.8S, ITS2 and 28SrDNA. The splits are well supported, with only posterior probabilities below 1 shown. The tree generally matches results based on recent phylogenomic work (Bond et al. 2014; Fernández et al. 2018). The sequence information for this phylogeny was generated by sequencing long PCR amplicons on a Nanopore third generation sequencer. The inset in the upper right shows interspecific and intraspecific pairwise genetic distances for 16 endemic Hawaiian *Tetragnatha* species for COI on the X-axis and the ribosomal cluster on the Y-axis. Long ribosomal sequences show a comparable taxonomic resolution to the widely used COI. Modified from Krehenwinkel et al. 2019a

**Supplementary Table 1** Useful primer sets for various applications of amplicon sequencing-based spider barcoding

| **Locus** | **F primer** | **F primer sequence 5' to 3'** | **R primer** | **R primer sequence 5' to 3'** | **Amplicon length** | **Description/application** |
| --- | --- | --- | --- | --- | --- | --- |
| COI | ArF1^1^ | GCNCCWGAYATRGCNTTYCCNCG | Fol-degen-rev^2^ | TANACYTCNGGRTGNCCRAARAAYCA | 467 | barcoding |
| COI | mlCOIintF^3^ | GGWACWGGWTGAACWGTWTAYCCYCC | Fol-degen-rev^2^ | TANACYTCNGGRTGNCCRAARAAYCA | 365 | barcoding |
| 16SrDNA | 16SF2^4^ | AATYCAACATCGAGGTCGCAA | 16SR2^4^ | TRACYGTRCWAAGGTAGCAT | 371 | barcoding |
| 12SrDNA | 12SF1^4^ | NCHACTWTGTTACGACTT | 12SR1^4^ | AMTAGGATTAGATACCCT | 424 | barcoding |
| Cytochrome B | CB3degB^4,5^ | GAGGDGCHACHGTWATYACHAA | CB4deg^4,5^ | RAARTATCATTCDGGTTGRATRTG | 406 | barcoding |
| 18SrDNA | 18s_2F^6^ | AACTTAAAGRAATTGACGGA | 18s_4R^6^ | CKRAGGGCATYACWGACCTGTTAT | 351 | barcoding |
| 18SrDNA | SSU_FO4^7^ | GCTTGTCTCAAAGATTAAGCC | SSU_R22^7^ | GCCTGCTGCCTTCCTTGGA | 421 | barcoding |
| 28SrDNA | 28s_3F^6^ | TTTTGGTAAGCAGAACTGGYG | 28s_4R^6^ | ABTYGCTACTRCCACYRAGATC | 363 | barcoding |
| ITS2 | 5.8S3F^4^ | ATCACTHGGCTCRYGGRTCGATG | 28S2R^4^ | TTCTTTTCCTCCSCTHANTDATATGC | 436 | barcoding |
| Histone H3 | H3aF^8^ | ATGGCTCGTACCAAGCAGACVGC | H3aR^8^ | ATATCCTTRGGCATRATRGTGAC | 374 | barcoding |
| ZBJ (COI) | ZBJ-ArtF1c^9^ | AGATATTGGAACWTTATATTTTATTTTTGG | ZBJ-ArtR2c^9^ | WACTAATCAATTWCCAAATCCTCC | 211 | gut content |
| 16SrDNA_gut | 16SF_885^10^ | ARGACGAGAAGACCCYATA | 16SR_1086^10^ | ATWACGCTGTTATCCCYAA | 201 | gut content |
| 18SrDNA_gut | 18SF_1332^10^ | AGCTCTTTCTYGATTCRGTGGGT | 18SR_1628^10^ | CACAGACCTGTTATTGCTCAA | 296 | gut content |
| 18SrDNA_gut | 18SF_1392^10^ | GTCTGGTTRATTCCGRTAACGAA | 18SR_1628^10^ | CACAGACCTGTTATTGCTCAA | 236 | gut content |
| 28SrDNA_gut | 28SF_788^10^ | CGTCTTGAAACACGGACCAA | 28SR_1062^10^ | GWCCTCCATCAGGGTTTCCC | 274 | gut content |
| nuclear rDNA | 18S_F4^11^ | GGCTACCACATCYAARGAAGGCAGCAG | 28S_R8^11^ | TCGGCAGGTGAGTYGTTRCACAYTCCT | variable; *ca.* 4200 in spiders | long-read barcoding |
| Minibar (COI) | Uni-MinibarF1^12^ | TCCACTAATCACAARGATATTGGTAC | Uni-MinibarR1^12^ | GAAAATCATAATGAAGGCATGAGC | 176 | mini-barcode |

^1^Gibson et al. (2014), ^2^Yu et al. (2012), ^3^Leray et al. (2013), ^4^Krehenwinkel et al. 2018, ^5^Barraclough et al. (1999), ^6^Machida and Knowlton (2012), ^7^Fonseca et al. (2010), ^8^Colgan et al. (1998), ^9^Zeale et al. (2011), ^10^Krehenwinkel et al. (2019c), ^11^Krehenwinkel et al. (2019a), ^12^Meusnier et al. (2008)

**Supplementary Table 2** Published spider genomes

| **Common Name** | **Latin Name** | **Sequencing Method** | **Reference/Genbank Accession** |
| --- | --- | --- | --- |
| House spider | *Parasteatoda tepidariorum* | Illumina (PE, MP, Chicago) | Schwager et al. 2017  GCA_000365465.2 |
| African social velvet spider | *Stegodyphus mimosarum* | Illumina (PE, MP) | Sanggaard et al. 2014  GCA_000611955.2 |
| African social velvet spider | *Stegodyphus dumicola* | Illumina (PE), PacBio | Liu et al. 2019 |
| Brazilian white-knee tarantula | *Acanthoscurria geniculata* | Illumina (PE, MP) | Sanggaard et al. 2014  GCA_000661875.1 |
| Golden orb-weaver | *Nephila clavipes* | Illumina (PE, MP) | Babb et al. 2017  GCA_002102615.1 |
| Brown recluse spider | *Loxosceles reclusa* | Illumina (PE, MP) | GCA_001188405.1 |
| Western black widow | *Latrodectus hesperus* | Illumina (PE, MP) | GCA_000697925.1 |
| Pond wolf spider | *Pardosa pseudoannulata* | Illumina (PE, MP, 10x Genomics), Pacbio | Yu et al. 2019  GCA_008065355.1 |
| Woodlouse hunting spider | *Dysdera silvatica* | Illumina (PE, MP), Pacbio, Nanopore | Sánchez-Herrero et al. 2019  GCA_006491805.1 |

**References**

Babb PL, Lahens NF, Correa-Garhwal SM, Nicholson DN, Kim EJ, Hogenesch JB, Kuntner M, Higgins L, Hayashi CY, Agnarsson I, Voight BF (2017) The *Nephila clavipes* genome highlights the diversity of spider silk genes and their complex expression. Nat Genet 49:895-903.

Barraclough TG, Hogan JE, Vogler AP (1999) Testing whether ecological factors promote cladogenesis in a group of tiger beetles (Coleoptera: Cicindelidae). P Roy Soc Lond B Bio 266:1061-1067.

Bond JE, Garrison NL, Hamilton CA, Godwin RL, Hedin M, Agnarsson I (2014) Phylogenomics resolves a spider backbone phylogeny and rejects a prevailing paradigm for orb web evolution. Curr Biol 24:1765-1771.

Colgan DJ, McLauchlan A, Wilson GDF, Livingston SP, Edgecombe GD, Macaranas J, Cassis G, Gray MR (1998) Histone H3 and U2 snRNA DNA sequences and arthropod molecular evolution. Aust J Zool 46:419-437.

Fernández R, Kallal RJ, Dimitrov D, Ballesteros JA, Arnedo MA, Giribet G, Hormiga G (2018) Phylogenomics, diversification dynamics, and comparative transcriptomics across the spider tree of life. Curr Biol 28:1489-1497.

Fonseca VG, Carvalho GR, Sung W, Johnson HF, Power DM, Neill SP, Packer M, Blaxter ML, Lambshead PJD, Thomas WK, Creer S, (2010) Second-generation environmental sequencing unmasks marine metazoan biodiversity. *Nat Commun*. https://doi.org/10.1038/ncomms1095

Gibson J, Shokralla S, Porter TM, King I, van Konynenburg S, Janzen DH, Hallwachs W, Hajibabaei M (2014) Simultaneous assessment of the macrobiome and microbiome in a bulk sample of tropical arthropods through DNA metasystematics. P Natl Acad Sci USA 111:8007-8012.

Kennedy S, Lim JY, Clavel J, Krehenwinkel H, Gillespie RG (2019) Spider webs, stable isotopes and molecular gut content analysis: Multiple lines of evidence support trophic niche differentiation in a community of Hawaiian spiders. Funct Ecol 33:1722-1733.

Krehenwinkel H, Kennedy SR, Adams SA, Stephenson GT, Roy K, Gillespie RG (2019c)^[[1]](#footnote-1)^ Multiplex PCR targeting lineage‐specific SNPs: A highly efficient and simple approach to block out predator sequences in molecular gut content analysis. Methods Ecol Evol 10:982-993.

Krehenwinkel H, Kennedy S, Pekár S, Gillespie RG (2017b) A cost‐efficient and simple protocol to enrich prey DNA from extractions of predatory arthropods for large‐scale gut content analysis by Illumina sequencing. Methods Ecol Evol 8:126-134.

Krehenwinkel H, Kennedy SR, Rueda A, Lam A, Gillespie RG (2018) Scaling up DNA barcoding – Primer sets for simple and cost efficient arthropod systematics by multiplex PCR and Illumina amplicon sequencing. Methods Ecol Evol 9:2181-2193.

Krehenwinkel H, Pomerantz A, Henderson JB, Kennedy SR, Lim JY, Swamy V, Shoobridge JD, Graham N, Patel NH, Gillespie RG, Prost S (2019a) Nanopore sequencing of long ribosomal DNA amplicons enables portable and simple biodiversity assessments with high phylogenetic resolution across broad taxonomic scale. GigaScience. https://doi.org/10.1093/gigascience/giz006

Krehenwinkel H, Wolf M, Lim JY, Rominger AJ, Simison WB, Gillespie RG (2017a) Estimating and mitigating amplification bias in qualitative and quantitative arthropod metabarcoding. Sci Rep-UK. https://doi.org/10.1038/s41598-017-17333-x

Leray M, Yang JY, Meyer CP, Mills SC, Agudelo N, Ranwez V, Boehm JT, Machida RJ (2013) A new versatile primer set targeting a short fragment of the mitochondrial COI region for metabarcoding metazoan diversity: Application for characterizing coral reef fish gut contents. Front Zool. https://doi.org/10.1186/1742-9994-10-34

Liu S, Aageaard A, Bechsgaard J, Bilde T (2019) DNA methylation patterns in the social spider, *Stegodyphus dumicola*. Genes. https://doi.org/10.3390/genes10020137

Machida RJ, Knowlton N (2012) PCR primers for metazoan nuclear 18S and 28S ribosomal DNA sequences. PLoS One. https://doi.org/10.1371/journal.pone.0046180

Meusnier I, Singer GA, Landry JF, Hickey DA, Hebert PD, Hajibabaei M (2008) A universal DNA mini-barcode for biodiversity analysis. BMC Genomics. https://doi.org/10.1186/1471-2164-9-214

Sánchez-Herrero JF, Frías-López C, Escuer P, Hinojosa-Alvarez S, Arnedo MA, Sánchez-Gracia A, Rozas J (2019) The draft genome sequence of the spider *Dysdera silvatica* (Araneae, Dysderidae): A valuable resource for functional and evolutionary genomic studies in chelicerates. GigaScience. https://doi.org/10.1093/gigascience/giz099

Sanggaard KW, Bechsgaard JS, Fang X, Duan J, Dyrlund TF, Gupta V, Jiang X, Cheng L, Fan D, Feng Y, Han L (2014) Spider genomes provide insight into composition and evolution of venom and silk. Nature Commun. https://doi.org/10.1038/ncomms4765

Schwager EE, Sharma PP, Clarke T, Leite DJ, Wierschin T, Pechmann M, Akiyama-Oda Y, Esposito L, Bechsgaard J, Bilde T, Buffry AD (2017) The house spider genome reveals an ancient whole-genome duplication during arachnid evolution. BMC Biology. https://doi.org/10.1186/s12915-017-0399-x

Yu DW, Ji Y, Emerson BC, Wang X, Ye C, Yang C, Ding Z (2012) Biodiversity soup: Metabarcoding of arthropods for rapid biodiversity assessment and biomonitoring. Methods Ecol Evol 3:613-623.

Yu N, Li J, Liu M, Huang L, Bao H, Yang Z, Zhang Y, Gao H, Wang Z, Yang Y, Van Leeuwen T (2019) Genome sequencing and neurotoxin diversity of a wandering spider *Pardosa pseudoannulata* (pond wolf spider). bioRxiv. https://doi.org/10.1101/747147

Zeale MR, Butlin RK, Barker GL, Lees DC, Jones G (2011) Taxon‐specific PCR for DNA barcoding arthropod prey in bat faeces. Mol Ecol Resour 11:236-244.

1. To avoid confusion, we retain the same lettering scheme used in the main paper even though Krehenwinkel et al. 2019b (Krehenwinkel, Pomerantz and Prost 2019) is not cited in the Supplement. [↑](#footnote-ref-1)
